# The association of knowledge, attitudes and behaviours related to salt with 24-hour urinary sodium excretion

**DOI:** 10.1186/1479-5868-11-47

**Published:** 2014-04-04

**Authors:** Mary-Anne Land, Jacqui Webster, Anthea Christoforou, Claire Johnson, Helen Trevena, Frances Hodgins, John Chalmers, Mark Woodward, Federica Barzi, Wayne Smith, Victoria Flood, Paul Jeffery, Caryl Nowson, Bruce Neal

**Affiliations:** 1 The George Institute for Global Health, Sydney Medical School, The University of Sydney, PO BOX M201, Missenden Road, Camperdown, Sydney, NSW, Australia; 2 Royal Prince Alfred Hospital, Sydney, New South Wales, Australia; 3 New South Wales Health, Sydney, Australia; 4 The Sydney University of Sydney and St Vincent’s Hospital, Sydney, Australia; 5 Deakin University, Melbourne, Australia

**Keywords:** Salt, Urinary sodium excretion, Knowledge, Attitude, Behaviour

## Abstract

**Aim:**

Salt reduction efforts usually have a strong focus on consumer education. Understanding the association between salt consumption levels and knowledge, attitudes and behaviours towards salt should provide insight into the likely effectiveness of education-based programs.

**Methods:**

A single 24-hour urine sample and a questionnaire describing knowledge, attitudes and behaviours was obtained from 306 randomly selected participants and 113 volunteers from a regional town in Australia.

**Results:**

Mean age of all participants was 55 years (range 20–88), 55% were women and mean 24-hour urinary salt excretion was 8.8(3.6) g/d. There was no difference in salt excretion between the randomly selected and volunteer sample. Virtually all participants (95%) identified that a diet high in salt can cause serious health problems with the majority of participants (81%) linking a high salt diet to raised blood pressure. There was no difference in salt excretion between those who did 8.7(2.1) g/d and did not 7.5(3.3) g/d identify that a diet high in salt causes high blood pressure (p = 0.1). Nor was there a difference between individuals who believed they consumed “too much” 8.9(3.3) g/d “just the right amount” 8.4(2.6) g/d or “too little salt” 9.1(3.7) g/d (p = 0.2). Likewise, individuals who indicated that lowering their salt intake was important 8.5(2.9) g/d vs. not important 8.8(2.4) g/d did not have different consumption levels (p = 0.4).

**Conclusion:**

The absence of a clear association between knowledge, attitudes and behaviours towards salt and actual salt consumption suggests that interventions focused on knowledge, attitudes and behaviours alone may be of limited efficacy.

## Background

The burden of non-communicable diseases constitutes a major public health challenge worldwide. Raised blood pressure is the leading risk factor for the global burden of disease, estimated to cause 9 · 4 million deaths every year—more than half of the 17 million deaths a year attributed to cardiovascular disease [1]. Decreasing dietary salt intake from the estimated global level of 9–12 grams per day(g/d) [2] to the recommended level of less than 6 g/d [3] would have a significant impact on blood pressure levels and cardiovascular disease, preventing up to 2.5 million deaths due to heart attacks and stroke worldwide each year [4]. Consequently programs to reduce population salt intake have been identified as a cost effective action that should be undertaken immediately to produce accelerated results in terms of lives saved, disease prevented and costs avoided [5].

The primary source of salt in the diets of developed countries is processed foods, with discretionary salt accounting for a larger proportion of the intake in many low and middle income countries [2]. Social and cultural factors, along with population age, educational level and average income are primary determinants of dietary behaviours but can be difficult to modify in the short term [6]. Population knowledge, attitudes and behaviours, on the other hand, are thought to influence salt consumption and are considered modifiable mediating factors that are amenable to change [7]. As such, salt reduction efforts often include interventions to raise consumer awareness. The objective of the present study was to describe the relationship between salt consumption levels and knowledge, attitudes and behaviours towards salt so as to provide insight into the likely effectiveness of salt reduction efforts based primarily upon health promotion and education.

## Methods

Data were collected from a cross–sectional survey conducted in Lithgow, a regional town with a residential population of about 20,160 [8] and a mean socio-economic index for area disadvantage score of 924.2 [9]. The average SEIFA score for Australia is 1000 and ranges between 700 and 1200 in the most advantaged and disadvantaged areas. Lithgow is located 140 kilometres west of the Sydney, New South Wales Australia. Permission to undertake the study was obtained from the Lithgow City Council and the project was approved by the University of Sydney Human Research Ethics Committee. The methods for participant selection and study conduct have been published elsewhere [10].

### Participant selection and recruitment

Individuals aged 20 years or above who were resident in Lithgow and listed on the electoral roll were eligible for inclusion in the study. Potential participants were required to provide written informed consent for enrolment to proceed. There was no exclusion based on illness, use of medications or any other aspect of demography or personal history. Recruitment was done by random sampling from the electoral roll and by asking for volunteers when random sampling delivered insufficient numbers.

*Random sampling* was done by selecting individuals at random from the electoral roll. The electoral roll provided the name and address of each potential participant with electronic databases searched to identify corresponding telephone numbers. Potential participants were first mailed invitations to take part in the survey, with an explanation of the purpose of the study, a participant information sheet and a consent form provided. These individuals were then contacted by telephone to determine their willingness to participate and to schedule an interview time. Where a telephone number could not be obtained, the home address was visited by a member of the research team and willingness to participate was discussed face-to-face. The response rate was poor (16%), however, a number of non-randomly selected volunteers were therefore included.

*Volunteer sampling* was done by offering participation in the study to individuals at two local shopping centres over several weeks. An information booth was established where those interested could seek further information about participation and arrange a visit by a member of the study team. Aside for the initial contact method the study was otherwise done in an identical manner for the random and volunteer samples.

### Data collection

Data collection comprised interviewer-administered questionnaires about demographics and knowledge, attitudes and behaviours related to salt, followed by a physical examination and a single 24-hour urine collection. The first set of these was done at the time of the visit with the urine collection scheduled to be completed within the next three days. The questions about knowledge, attitudes and behaviours were adapted from the World Health Organization/Pan American Health Organization protocol for population level sodium determination [11]. The questionnaire contained nine questions; four related to knowledge of personal consumption, recommended daily intake and possible harmful effects of salt and five assessing attitudes and behaviours to lowering salt intake. The participants answered on a range of different scales such as “rarely, sometimes, often”, “yes, no” and “too much, just the right amount, too little”.

The physical examination comprised measurement of body weight (using calibrated Tanita HD-357 portable electronic scales (USA) and height (using a calibrated portable stadiometer Wedderburn WS-HRP model (Australia)) to the nearest 0.1 kg and 0.1 cm respectively. Body mass index (weight (kg)/height(m^2^)) was then calculated. Blood pressure was measured using a manual inflation blood pressure monitor (A&D UA-&704) in triplicate, according to the American Heart Association protocol [12].

A single 24-hour urine collection was obtained with the first voided urine upon waking on the day of collection being discarded and participants then collecting all voided urine up to, and including, the first void the following morning. Participants were instructed to keep collected samples inside cooler bags provided and stored in a cool, dark place until completion when a research assistant was contacted to collect the sample. The times at the beginning and the end of urine collection were recorded. The urine volume was noted and the urinary sodium concentration in an aliquot was measured by ion-selective electrode with the buffered kinetic Jaffe reaction without deproteinisation used for assay of urine creatinine (Cobas Integra 400). Suspected inaccurate urine collections (i.e. urinary creatinine < 4.0 mmol/day for women, or < 6.0 mmol/day for men, or a 24-hour urine collection of < 500 ml for either sex) and extreme outliers for urinary creatinine (i.e. > 3 standard deviations from the mean for either sex) were excluded [13,14]. For each individual, the 24-hour sodium excretion value (mmol/day) was calculated as the concentration of sodium in the urine (mmol/L) multiplied by the urinary volume (L/day). The conversion from sodium (Na) to salt (NaCl) was made by multiplying the sodium value by 2.542 (NaCl(g) = Na(g) × 2.542).

In recognition of the inconvenience associated with the study procedures, participants that completed all components of the survey were provided with an AUD$40 shopping voucher.

### Statistical analysis

The baseline characteristics of the sample were summarised as proportions and means (standard deviation or range). All primary analyses were done on the combined sample of randomly selected individuals and volunteers with subsidiary analyses done to check that the findings were similar between the two populations. The associations of knowledge, attitudes and behaviours with 24-hour urinary sodium excretion were investigated by making comparisons using linear regression, however because two covariates (sex and education) are not continuous the term general linear model is applied which incorporates both categorical and continuous variables. We first made crude estimates and then estimates adjusted for age, sex, body mass index and highest level of education. The adjusted estimates constituted the primary results with the first three covariates included on the basis of their observed association with salt consumption, and the latter on the basis of a strong rationale for potential confounding by this factor. P values <0.05 were deemed significant. There was no formal adjustment made for the multiplicity of testing but all findings were interpreted in light of the number of comparisons made and the broader pattern of findings across the data. Statistical analyses were conducted using SPSS for Windows (Version 21, SPSS Inc, Chicago, IL).

## Results

**Figure 1:**
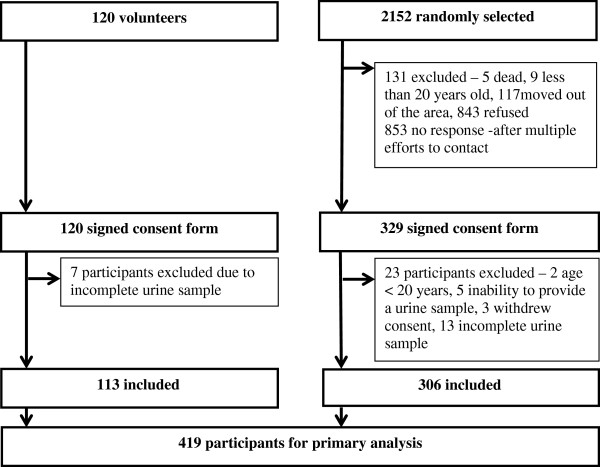
Recruitment of population sample.

Of 2,152 individuals selected by random sampling of the electoral roll, 853 (40%) were un-contactable after multiple attempts, 126 (5.8%) were ineligible because they had moved out of the study area, 5 (0.2%) had died and 843 (39%) declined to participate. The remaining 329 individuals (response rate 16%) comprise the ‘random’ sample from which 23 individuals were subsequently excluded because of suspected incomplete urine collections (n = 13), omitted on the basis that they withdrew consent (n = 3), were unable to provide a urine sample due to medical conditions (n = 5) or were subsequently found to be less than 20 years old (n = 2). The volunteer sample comprised 120 individuals from which seven were excluded because of suspected incomplete urine collections (Figure 1). All 419 participants with valid urine samples completed the questionnaire about knowledge, attitudes and behaviours. The characteristics of the random and volunteer samples were moderately different in a number of regards, including age, proportion using tobacco, alcohol use, self-reported hypertension and use of any prescription medication (Table 1) but these did not translate into detectable differences in the observed associations.

**Table 1:** Characteristics of participants

	**Total sample (n = 419)**	**Random sample (n = 306)**	**Volunteer sample (n = 113)**
Age, years (mean, range)	55.4(20–88)	57.6(20–88)	49.3(20–84)
Female (%)	55	53	62
Height, cm (mean, SD)	167.5(9.6)	167.5(9.9)	167.7(8.7)
Weight, kg (mean, SD)	82.4(18.1)	81.8(17.1)	83.9(21)
Body Mass Index, kg/m^2^(mean, SD)	29.3(5.6)	29.1(5.2)	29.8(6.6)
Systolic Blood Pressure, mmHg (mean, SD)	125.8(19.2)	126.6(17.7)	124(22.6)
Diastolic Blood Pressure, mmHg (mean, SD)	78.7(12)	78.7(10.8)	78.9 (16.7)
Education			
-Secondary or below (%)	63	65	59
-Tertiary (%)	27	26	33
-Postgraduate (%)	10	9	8
Health Status			
-Very good (%)	50	50	49
-Good (%)	28	29	24
-Fair (%)	22	21	27
Smoking status			
-Current (> 1/day for last year) (%)	12	8	22
-Ever (%)	44	41	53
Alcoholic consumption (time since last consumption)			
One week or less (%)	52	62	43
> One week < 12 months (%)	27	32	34
12 months or more (%)	11	4	13
Never (%)	10	2	10
Disease history			
-High blood pressure (%)	38	41	29
-Low blood pressure (%)	15	15	14
-High cholesterol (%)	36	38	30
-Heart attack (%)	7	8	4
-Stroke (%)	3	4	2
-Angina (%)	6	7	4
-Diabetes (%)	10	10	7
Prescription Medication Use			
-Antihypertensive (%)	22	22	20
-Lipid lowering (%)	16	16	18
-Aspirin (%)	6	8	3
-Glucose lowering (%)	6	5	9
-Any prescription medication (%)	62	65	53

### 24 hour urinary salt excretion levels

The mean 24-hour urinary salt excretion amongst the 419 participants was 8.8 (standard deviation, 3.6) g/d. The proportion exceeding the World Health Organization recommended maximum level of 5 g/d was 87%, the proportion exceeding the Australian Government recommended maximum level of 6 g/d was 78%, and proportion consuming the Australian Government suggested dietary target of 4 g/d or less was just 6%. Urinary salt excretion was significantly higher in men compared to women (p < 0.001), there was an inverse association between daily salt excretion and age (p = 0.007) and a positive association between salt excretion and BMI (p < 0.001). There was no significant association between highest level of education and salt consumption (p = 0.10).

### Knowledge, attitudes and behaviours towards salt

Almost all participants (95%) identified that a diet high in salt can cause serious health problems with the majority of participants (81%) linking a high salt diet to raised blood pressure. One half (50%) of the participants described themselves as consuming “the right amount” of salt although less than one fifth (18%) recognised the recommended Australian upper daily intake for dietary salt (6 g/d). Just over two thirds (63%) of participants reported taking action on a regular basis to control their salt intake. Of those who reported taking action to lower their salt intake, participants did so by avoiding processed foods (44%), using low salt alternatives (34%), and checking labels (30%) or by using spices other than salt (29%). Actions to control salt intake were more common in women than men (p = 0.04) but otherwise there were no differences detected between the responses of the sexes, different age groups, individuals with different levels of body mass index or individuals with different levels of education (all p > 0.05).

### Associations of knowledge, attitudes and behaviours with 24-hour urinary salt excretion

There was no evidence of an association between any measure of knowledge, attitudes or behaviours and urinary salt excretion either before (all p > 0.17) or after (all p > 0.10) adjustment for age, sex, body mass index and highest level of education (Table 2). The findings were the same in subsidiary analyses restricted to just the randomly selected individuals or just the volunteer sample.

**Table 2:** Association between knowledge, attitudes and behaviours and urinary salt excretion (grams per day)

			**Adjusted estimate**^**+**^	
		**%**	**Mean (g/d)**	**P value**
**Knowledge**				
Max salt consumption recommendation	<10 g	10	8.5	0.81
	<6 g	18	8.4	
	<4 g	31	8.9	
	<2 g	41	8.7	
Does a high salt diet cause health problems?	Yes	95	8.7	0.10
	No	5	7.5	
If yes, what problems? (Raised Blood Pressure)	Yes	81	8.7	0.89
	No	19	8.6	
**Attitudes**				
How much salt do you think you consume?	Too much	28	8.9	0.15
	Right amount	50	8.4	
	Too little	22	9.1	
How important to you is lowering salt in your diet?	Not important	36	8.5	0.39
	Important	64	8.8	
**Behaviour**				
Add salt to food at table	Rarely	52	8.9	0.32
	Sometimes	27	8.6	
	Always	21	8.3	
Add salt when cooking	Rarely	54	8.7	0.99
	Sometimes	27	8.7	
	Always	19	8.7	
Take regular action taken to control your salt intake?**	Yes	63	8.8	0.25
	No	37	8.5	
If yes, what?				
-Avoid processed foods	Yes	44	8.6	0.37
	No	56	8.6	
-Check labels	Yes	30	8.8	0.17
	No	70	8.5	
-Buy low salt alternatives	Yes	34	9.0	0.10
	No	66	8.5	
-Use spices	Yes	29	8.6	0.69
	No	71	8.7	
-Avoid eating out	Yes	20	8.8	0.65
	No	80	8.7	

**+**Adjusted for possible confounding effects of age, sex, education and body mass index.

**Participants could be taking more than one action to reduce salt intake.

## Discussion

We identified no association between consumer knowledge, attitudes and behaviours relating to salt and actual levels of salt intake. Furthermore, while most participants identified that salt is detrimental to health, and large numbers reported actions to address their salt consumption, the great majority of this sample of the Australian population continued to consume salt at a level above national and international recommendations.

The levels of knowledge, attitudes and behaviours of the participants are similar to those reported in a prior survey of a national sample of Australian consumers done in 2007 [15,16] and a more recent study conducted in metropolitan Melbourne [17]. Both these studies found that the majority of participants were aware of the harmful effect of salt on health but also showed that few were able to identify the recommended upper daily intake and revealed limited knowledge of the main foods that contribute to salt in the diet. Those Australian studies are also likely to have enrolled participants with comparable average levels and patterns of salt consumption to those reported here. Similar surveys have also been done for populations in five countries in the Americas (Argentina, Canada, Chile, Costa Rica, and Ecuador) [18] with similar findings, suggesting that the results found in this most recent Australian study are not atypical.

Our examination of the association between knowledge, attitudes and behaviours and actual levels of salt consumption is novel and raises questions about the likely capacity of purely educational interventions to change individual or community salt consumption levels. Given the high levels of knowledge and positive attitudes to salt reduction recorded in our survey, the most likely explanation for high salt consumption levels across the community is that there are many barriers to changing behaviour. The adverse nature of the food environment which comprises heavily advertised, low-cost foods high in salt, and without adequate labelling of salt levels on packaging, is likely to be a key factor inhibiting reductions in salt consumption amongst even well-informed individuals [19].

In contrast, several intervention trials have demonstrated effects of nutrition education on salt consumption in selected groups of individuals [20-22]. The reason why these trials had positive effects is most likely because of the high intensity of the interventions applied, which typically included multiple one-on-one consultations with participants, small group activities or provision of reduced salt foodstuffs. While clearly effective, this type of approach is not feasible at the population level because of the resources required [23] and the observed benefits cannot reasonably be generalised to community settings where average exposure of individuals to educational interventions targeting salt is so much less. It is of note that in the UK, Japan and Finland, where population-wide salt reduction has been achieved, community education was underpinned by programs that changed the average salt levels in key foods, used salt warning labels to reinforce the health promotion messages and/or altered the broader food environment in some other way [24-26].

This study benefits from assessment of salt consumption based upon 24-hour urine collections and the use of standardised questions about knowledge, attitudes and behaviours related to salt. While the questionnaires have not undergone rigorous test-retest evaluation these types of survey outcomes are widely considered valid within the field. The cross-sectional design means that there remains some uncertainty about cause and effect in the results observed although when considered in the broader context, the findings are not inconsistent with the existing literature in this field. Standard checks for completeness of the specimens based upon urine volume and urine creatinine excretion were done but it remains likely that some urine samples were over-collections and others were under-collections. Para-aminobenzoic acid (PABA) has been used as a marker for completeness in overseas studies [27], but it is not without limitations [28] and currently not approved for use in Australia [29]. We believe that any errors in the included data are likely to be random and while this should not affect the mean group level estimates of salt intake, it could have reduced the power to detect associations between exposures and outcomes. In addition, because only one estimate was collected for each individual, and because there is significant within person variation in salt consumption from day to day, the variability of salt consumption within the population is likely to have been over-estimated. In turn, this means that the estimated proportions of individuals meeting the various salt targets (all of which lie below the population mean) are likely to have been over-estimated. It is likely that social approval bias [30,31] resulted in more socially desirable responses but we do not believe this is likely to have happened differentially across population subgroups and think it is unlikely to have substantively confounded our results. The survey was restricted to one regional town with a below average socio-economic index for area (SEIFA) score which raises concerns about the generalizability of the data. The SEIFA score is derived from attributes that reflect disadvantage such as low income, low educational attainment, high unemployment and jobs in relatively unskilled occupations [9]. However, comparable levels of knowledge and reported behaviours in other Australian surveys [32-38] using a range of different instruments [15-17] suggest that the findings are likely also to be valid outside of Lithgow. The constancy of the results across unadjusted and confounder-adjusted models, and in each of the study sub-populations, also argues for a fairly robust finding.

In conclusion, many of the participants surveyed were knowledgeable about the adverse effects of salt and reported efforts to ameliorate the risks caused by excess salt consumption. The persisting high levels of salt consumption across the population and the absence of any detectable association of knowledge levels with actual salt consumption are a significant concern. These data strongly suggest that education alone will be ineffective in reducing population salt consumption levels and that education programs must be supported by interventions that change the food environment in ways that encourage population-wide behaviour change. The food industry will play a central role in achieving population-wide salt reduction through the process of food reformulation, which has a key advantage over targeted behavioural and education interventions in that it can be delivered and sustained at scale [39] This is by no means a message unique to salt reduction efforts, with programs targeting obesity and alcohol consumption identifying similar limitations for programs based purely on education [40,41].

## Competing interests

B.N. is the Chairman of the Australian Division of World Action on Salt and Health and J.W. is Director of the World Health Organization Collaborating Centre on Population Sodium Reduction.

## Authors’ contributions

ML contributed to study concept and design, data collection, analysis and interpretation of the data, drafting the article and final version of the article. AC, CJ, HT, FH contributed to the analysis and interpretation of data. JW, JC, FB, VF, PJ, MW, WS and CN contributed to the conception and study design and revision of content to be published. BN contributed to the conception and study design, analysis and interpretation of data, drafting of the article and revising critically. All authors read and approved the final manuscript.
